# Pain State Classification of Stiff Knee Joint Using Electromyogram for Robot-Based Post-Fracture Rehabilitation Training

**DOI:** 10.3390/s25165142

**Published:** 2025-08-19

**Authors:** Yang Zheng, Dimao He, Yuan He, Xiangrui Kong, Xiaochen Fan, Min Li, Guanghua Xu, Jichao Yin

**Affiliations:** 1 School of Mechanical Engineering, Xi’an Jiaotong University, Xi’an 710049, China; ightout2023@gmail.com (D.H.); kongxr@stu.xjtu.edu.cn (X.K.); min.li@mail.xjtu.edu.cn (M.L.); xugh@mail.xjtu.edu.cn (G.X.); 2 State Industry-Education Integration Center for Medical Innovations, Xi’an Jiaotong University, Xi’an 710049, China; 3 TCM Rehabilitation Department, Honghui Hospital, Xi’an Jiaotong University, Xi’an 710001, China; he_yuan1995@163.com (Y.H.); xiaochenfan_hhyy@126.com (X.F.); 4 Xi’an Health School, Xi’an 710054, China; 83797727@163.com

**Keywords:** pain, range of motion, electromyogram, fracture, pattern recognition

## Abstract

Knee joint stiffness occurs and severely limits its range of motion (ROM) after facture around the knee. During mobility training, knee joints need to be flexed to the maximum angle position (maxAP) that can induce pain at an appropriate level in order to pull apart intra-articular adhesive structures while avoiding secondary injuries. However, the maxAP varies with training and is mostly determined by the pain level of patients. In this study, the feasibility of utilizing electromyogram (EMG) activities to detect maxAP was investigated. Specifically, the maxAP detection was converted into a binary classification between pain level three of the numerical rating scales (pain) and below (painless) according to clinical requirements. Firstly, 12 post-fracture patients with knee joint stiffness participated in Experiment I, with a therapist performing routine mobility training and EMG signals being recorded from knee flexors and extensors. The results showed that the extracted EMG features were significantly different between the pain and painless states. Then, the maxAP estimation performance was tested on a knee rehabilitation robot in Experiment II, with another seven patients being involved. The support vector machine and random forest models were used to classify between pain and painless states and obtained a mean accuracy of 87.90% ± 4.55% and 89.10% ± 4.39%, respectively, leading to an average estimation bias of 6.5° ± 5.1° and 4.5° ± 3.5°. These results indicated that the pain-induced EMG can be used to accurately classify pain states for the maxAP estimation in post-fracture mobility training, which can potentially facilitate the application of robotic techniques in fracture rehabilitation.

## 1. Introduction

Traffic accidents, sports activities, military training, natural disasters, and the increasing incidence of osteoporosis due to population aging have led to many fractures worldwide every year. Fracture treatment involves three major processes, i.e., reduction [1], fixation [2], and rehabilitation training [3]. Recently, a systematic review showed that early rehabilitation is effective in improving joint function among post-operative fracture patients and allows patients to return to daily life earlier [4]. In contrast to the reduction and fixation treatment that are usually performed in hospitals through surgery with good completion and controllability, post-operative rehabilitation training is typically conducted at home. Due to the lack of professional rehabilitation guidance, improper or insufficient training can lead to complications such as joint stiffness [5]. Multiple factors can cause joint stiffness, including joint swelling [6], intra-articular adhesions, joint contractures, fibrosis of peri-articular structures, and pain [5]. Joint stiffness affects the essential function of joints by limiting its range of motion (ROM) and hinders the rehabilitation process, resulting in negative impacts on quality of post-operative life [7].

In order to increase the ROM of joints with stiffness, the therapists need to assist patients in rotating the affected joint and maintaining the maximum angle position (maxAP) that should induce pain sensation at a proper level in the stiff joint, indicating that the adhesive tissues are split adequately, while avoiding possible secondary injuries. Repetitive mobility training to the maxAP can help to pull apart the adhesive intra-articular tissues and therefore is the key to improving rehabilitation outcomes. However, the maxAP increases with training and cannot be determined in advance. Instead, currently in clinical practice, the estimation of maxAP is highly dependent on the experience of therapists according to the pain level reported by patients during joint mobility training. Due to the lack of rehabilitation therapists, a majority of post-fracture patients with joint stiffness cannot receive proper treatment in time, not to mention repetitive and intensive training. Recently, the development of rehabilitation robotic techniques has provided a new approach for patients with motor dysfunction to perform motor rehabilitation training without the assistance of therapists [8,9]. The rehabilitation robots have the advantages of motion stability and repeatability, which enables high-intensity, repetitive training in order to achieve satisfactory rehabilitation outcomes. However, most currently available rehabilitation robots are designed for individuals with motor dysfunction caused by nervous system diseases such as stroke [9]. Although some exoskeleton robotic systems have also been used for rehabilitation after orthopedic surgery such as total knee arthroplasty [10], to the best of our knowledge, these robotic systems lack the ability to recognize the pain level at stiff joints of patients and therefore are incapable of helping patients rotate the affected joints to its maxAP autonomously. Although simple continuous passive motion devices can provide high-intensity repetitive training, intra-articular adhesive tissues remain unstretched, leading to poor training outcomes. Therefore, a robotic system specialized for post-fracture rehabilitation should have the ability to recognize the pain state at some specific level in the stiff joints in order to determine the maximum or optimal joint angle position for training. 

Pain is composed of various discomforts rather than a single entity [11]. Due to the complexity of pain sensation and its strong subjectivity, as well as the difficulty in accurately describing it in words, quantifying and assessing pain is a big challenge. The conventional methods of measuring pain level rely solely on patients’ self-report as the gold standard. The most used clinical assessment methods are based on pain assessment scales, including the visual analog scale [12], McGill Pain Questionnaire [13], and Numeric Pain Rating Scale [14]. Although these scale-based methods can assess pain to some extent, they have multiple limitations due to pain as a complex experience involving both physiological and emotional aspects. Moreover, these scale-based methods cannot be used in robotic systems to autonomously recognize pain states.

Although it is currently impossible to develop some kind of sensors to measure pain level directly, many researchers have been trying to explore the neurophysiological indicators of pain sensation to provide possible tools for objective and accurate pain measurement. In the field of cognitive neuroscience, researchers often use techniques such as electroencephalogram (EEG) and functional magnetic resonance imaging (fMRI) to detect pain-induced brain activities and their changes over time. Some researchers tried to establish an objective and effective pain measurement system by exploring the neural mechanisms of pain. For example, Huang et al. [15] developed a new method based on the relationship between laser-evoked potentials and pain, achieving precise pain sensing using single-trial data. By utilizing the advantage of fMRI with high spatial resolution, Marquand [16] has combined the whole-brain activation pattern and the supervised machine learning technique to predict thermal pain ratings. Similarly, Brown [17] verified that the support vector machine (SVM) model trained on fMRI data can assess pain in the absence of self-reporting, and a classification of 81% between painful and non-painful thermal stimuli was obtained. In terms of physiological measurements, researchers typically use signals such as skin conductance, skin temperature, heart rate, eye movements, and electromyography (EMG) to detect the response of the autonomic nervous system to pain stimulation, and they try to extract physiological indicators of pain sensation. A previous study has found that changes in heart rate and skin conductance levels are significantly correlated with stimulus intensity and participants’ pain ratings [18]. Pupil diameter was also demonstrated to reflect the intensity of painful stimuli [19]. Another study recorded the skin conductance and pupil diameter changes in response to the thermal pain stimuli of varying durations and temperatures and achieved the prediction of pain intensity in humans, albeit requiring a longer signal length of approximately 20 s [20]. Furthermore, the neuromuscular activity related to pain can also provide a possible tool for objective and accurate measurement of pain. For example, a previous EMG study [21] involving 19 healthy male subjects examined the effects of glutamate-induced jaw or neck muscle pain on their EMG activity. The results indicated that experimentally induced jaw muscle pain was associated with increased EMG activity in the subjects’ neck muscles. 

Although the current research on the neurophysiological or physiological characterization of pain provides various pain recognition methods that do not rely on patients’ self-reporting, the pain induced by thermal [20] or laser [15] stimuli under the experiment condition could be different from that induced by rotation of stiff joints. In addition, some studies have focused on the ability of classifying between the pain and non-pain state, which cannot satisfy the needs of identifying the pain state at some specific level for the mobility training of stiff joints. Although some other studies have demonstrated the correlation between the indicators and pain ratings, the ability of performing the pain recognition in real-time has not been proved [20]; or the device needed, for example, fMRI, is inapplicable for routine rehabilitation training [16]. In order to alleviate these issues, we explored the feasibility of using the EMG recordings to recognize the pain state during flexion of stiff knee joints after fractures around the knee in this study. The basic assumption was that the pain induced by flexion of stiff knee joints would cause an instinctive reaction of the extensors unconsciously contracting in order to resist further joint flexion, which could potentially contain the information reflecting the pain state. The knee joint was selected in this study mainly because the knee joint plays an important role in locomotion [22], and the morbidity of knee stiffness is high due to its complicated intra-articular structure. In short, EMG signals were recorded during the flexion of stiff knee joints until the pain reached a specific level needed in clinical application. The time/frequency domain and nonlinear EMG features were first analyzed in order to reveal whether the pain induced by rotation of stiff knee joints can affect the EMG activity. Then, based on the extracted EMG features, two classifiers were tested to identify the pain state at the specific level during the flexion of stiff knee joints and resultantly predict the maxAP. It should be noted that the proposed method is not trying to measure the pain directly. Instead, the indirect reaction of muscle contraction induced by pain is analyzed to vicariously sense the pain level. Our results showed that the pain state at some specific level caused by flexion of stiff knee joints can be predicted using the unconscious EMG activity, which provides a potential way for indirect vicarious sensing of pain to determine the maximum angle position autonomously for fracture rehabilitation robotic systems.

## 2. Method

### 2.1. Experiment Design

Nineteen subjects after fractures around the knee were recruited in this study. They had different degrees of limited ROM during knee flexion and were undergoing rehabilitation therapy at the Xi’an Honghui Hospital when they participated in the study. All participants signed informed consent approved by the Ethics Committee of Xi’an Honghui Hospital (Approval No. 202406018).

Two experiments were designed in this study. Since there were no previous studies showing that the pain induced by rotation of stiff joints can result in a change in EMG activity, the first experiment (Experiment I) was used to verify whether the pain sensation caused by the flexion of stiff knee joints can result in significantly varied EMG activities. This is the prerequisite of pain state classification using EMG signals. In the second experiment (Experiment II), the estimation of the maxAP was realized by solving a binary classification problem of the pain state, and the accuracy of maxAP estimation was tested with subjects wearing a previously developed knee exoskeleton rehabilitation robot [23]. Twelve subjects participated in Experiment I, and the other seven subjects participated in Experiment II (Table 1). In both experiments, the EMG signals from the musculus rectus femoris (MRF), musculus vastus medialis (MVM), musculus biceps femoris caput longum (MBF-CL), and musculus semitendinosus (MS) were recorded during the passive flexion of the knee joints. These muscles were selected mainly because they were the key superficial muscles to extend or flex the knee joint and have partially been used in a previous pain-related study [24]. The MRF and MVM are the extensors, while the other two are the flexors of the knee joint. Before the experiment, the skin surface covering the muscles were cleaned using an alcohol pad to decrease contact resistance, and two gel-based electrodes with a diameter of 1 cm were placed at each muscle belly with an inter-electrode distance of 3 cm. Bipolar EMG signals were amplified and acquired using a Biosignalsplux system (PLUX, Lisboa, Portugal) with a sampling rate of 2000 Hz. None of the subjects had ever participated in any experiments involving EMG recordings.

#### 2.1.1. Experiment I

**Figure 1:**
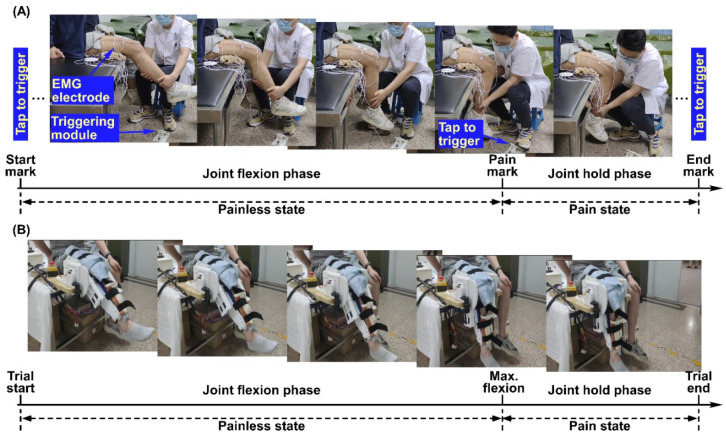
Experiment procedure and set-up for Experiment I (**A**) and Experiment II (**B**).

**Figure 2:**
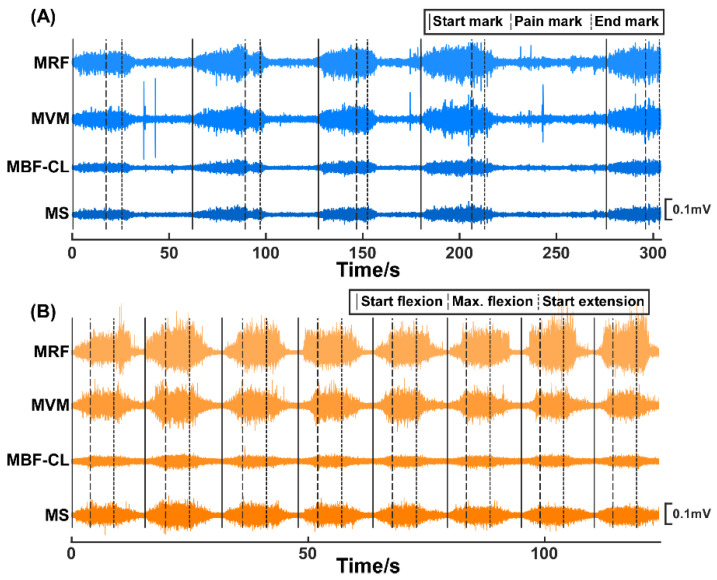
The original EMG signals of the MRF, MVM, MBF-CL, and MS muscles from two representative subjects participating in Experiment I (**A**) and Experiment II (**B**), respectively.

In Experiment I (Figure 1A), the routine joint mobility training was performed by an experienced therapist. A customized brake-like triggering module was connected to the Biosignalsplux system so that the therapist could send the event signal by treading on the triggering module. Within each trial, the therapist grasped subjects’ calf with his right hand, held subjects’ ankle with his left hand, and flexed the knee joint slowly. At the beginning of each trial, the therapist would tap on the triggering module to mark the start of the trial (termed the start mark) and immediately start flexing the knee joint. During knee joint flexion, subjects were requested to verbally report the pain level quantified using the numerical rating scale (NRS) when they felt the pain level varying. The therapist would stop flexing the knee joint further and tap on the triggering module (termed the pain mark) when the subjects reported pain level at three. Then, the knee joint was held at this maximum flexion angle position for approximately three seconds until the therapist tapped on the triggering module (termed the end mark) and started to extend the knee joint. Correspondingly, the period from the start mark to the pain mark was the joint flexion phase, and the period from the pain mark to the end mark was the joint hold phase. Each subject underwent 5 trials of training. Figure 2A shows the original EMG signals from a representative subject of Experiment I.

#### 2.1.2. Experiment II

In Experiment II (Figure 1B), a previously developed knee exoskeleton rehabilitation robot [23] was used to perform the mobility training. The robot can assist patients to flex and extend the knee joint within a preset range. After wearing the robot, the experimenter would first control the robot via a customized GUI to flex subjects’ knee joint slowly until they reported the NRS pain level at three. The corresponding joint angle was then set as the current maxAP for mobility training. Within each trial, the robot started from a comfortable extension position of subjects’ knee joints and assisted to flex to the maxAP. The robot would hold on at the maximum flexion angle for approximately 5 s and then started to extend to the initial position. Correspondingly, the period from the trial start to the timing of reaching the maxAP was the joint flexion phase and the period from the timing of reaching maxAP to the timing of starting extension was the joint hold phase. Each subject underwent 8 trials. The subjects were requested to report if the pain level did not reach pain level three of the NRS at the maxAP for the successive trials, and it turned out that none of the subjects reported a decreased pain level during the experiments. Figure 2B shows the original EMG signals from a representative subject of Experiment II. 

### 2.2. Data Processing

#### 2.2.1. Preprocessing

The original EMG signals were first band-pass-filtered between 20 Hz and 450 Hz and notch-filtered at 50 Hz using the zero-phase Butterworth filter. The filtered EMG signals were then segmented into five segments containing each signal from the start mark to the end mark (Experiment I) or eight segments containing each signal from the trial start to the timing of starting extension (Experiment II), corresponding to a complete trial. These EMG segments were used for further analysis, and the rest of EMG signals were discarded.

#### 2.2.2. Extraction of EMG Features

For a realistic robotic system for rehabilitation training, the pain state and the resultant maxAP is expected to be detected in real time. Therefore, the EMG signals were processed using a pseudo-online method. Specifically, EMG features were extracted within each 250-millisecond sliding window with a 125-millisecond step, and the pain state was identified for each sliding window. Twelve categories of commonly used time and frequency domain features and nonlinear features were extracted for each channel of EMG signals within each window. Although the EMG amplitude was mostly used to explore the muscle activity induced by pain [25,26], the features reflecting the frequency components of EMG activity were also used, mainly because a previous study [24] showed that pain can affect the motor unit discharge rate. In addition, the wavelet packet features and sample entropy were calculated, mainly considering the non-stationary and nonlinear property of the EMG signals, although they have not been used in previous pain-related studies. Given the EMG signal $x_{i}$ (i=1,2,…,N) and its corresponding power spectral density $P_{j}$ (j=1,2,…,N/2) with *N* as the sample length of each window, the features were calculated as follows [27]:

Root mean square

(1) $$RMS=\sqrt{\frac{\sum_{i=1}^{N}x_{i}^{2}}{N}}$$

Mean absolute value

(2) $$MAV=\frac{1}{N}\sum_{i=1}^{N}\left|x_{i}\right|$$

Variance

(3) $$\begin{array}{l} VAR=\frac{1}{N-1}\sum_{i=1}^{N}{\left(x_{i}-\bar{x}\right)}^{2}\\ \bar{x}=\frac{1}{N}\sum_{i=1}^{N}x_{i} \end{array}$$

Waveform length

(4) $$WL=\sum_{i=1}^{N-1}\left|x_{i+1}-x_{i}\right|$$

Zero crossings

(5) $$ZC=\sum_{i=1}^{N-1}f(i),f(i)=\left\{\begin{array}{l} 1,\;x_{i}x_{i+1}<0\;\mathrm{and}\;\left|x_{i}-x_{i+1}\right|>\varepsilon \\ 0,\;\mathrm{otherwise} \end{array}\right.$$

with ε = 0.005 mV being the threshold to reduce the noise-induced zero crossings. 

Number of slope sign change, SSC

(6) $$SSC=\sum_{i=2}^{N-1}f(i),f(i)=\left\{\begin{array}{l} 1,\;(x_{i}-x_{i+1})(x_{i}-x_{i-1})>0\;\mathrm{and}\;\left|x_{i}-x_{i+1}\right|>\varepsilon \;\mathrm{and}\;\left|x_{i}-x_{i-1}\right|>\varepsilon \\ 0,\;\mathrm{otherwise} \end{array}\right.$$

with ε = 0.005 mV being the threshold.

Mean frequency

(7) $$MNF=\frac{\sum_{j=1}^{N/2}f_{j}P_{j}}{\sum_{j=1}^{N/2}P_{j}}$$

Median frequency

(8) $$\sum_{i=1}^{MDF}P_{j}=\sum_{i=MDF}^{N/2}P_{j}$$

Wavelet packet

Wavelet transform is widely used to analyze the time/frequency characteristics of EMG signals, providing both time–domain and frequency–domain information at different scales. The wavelet packet transform (WPT) [28], built upon wavelet decomposition, is a signal decomposition and reconstruction algorithm based on a complete binary tree structure. Unlike the conventional wavelet decomposition that only splits the low-frequency sub-bands, WPT further decomposes both the low-frequency and high-frequency sub-bands, achieving higher time/frequency resolution and enabling more refined signal analysis. In this study, the Daubechies 4 (Db4) wavelet basis function was employed for wavelet packet decomposition, with a three-level decomposition applied to the EMG signals within each sliding window, resulting in 8 sub-bands in total. Then, three types of features were computed based on the coefficient of each sub-band, including the root mean square (WPT-RMS), variance (WPT-VAR), and the energy (WPT-Energy). 

Sample entropy

Sample entropy (SampEn) is a robust metric for quantifying the complexity and irregularity of time series data. It is widely used in various areas and evaluates the predictability of dynamic systems without requiring strict assumptions about data distribution. Compared with the approximate entropy, it eliminates self-comparisons for more accurate entropy estimation and only needs two parameters, including the embedded dimension *m* and the tolerance threshold *r*, resulting in reduced bias and improved parameter robustness. Its computing process is as follows:

With the embedded dimension *m* (*m* was set to 2 in this study), the vectors $X_{i}^{m}=[x_{i},\;x_{i+1},\;\ldots \;,x_{i+m-1}]$ (i=1,2,…,N−m+1) were reconstructed first. For each vector $X_{i}^{m}$, calculate the Chebyshev distance (maximum absolute difference) to all other vectors $X_{j}^{m}\;(j\neq i)$:

(9) $$d(X_{i}^{m},X_{j}^{m})=\underset{0\leq k\leq m-1}{\max}\left|x_{i+k}-x_{j+k}\right|$$

For each $X_{i}^{m}$, its similarity compared with the other vectors was calculated as [29]:

(10) $$B_{i}^{m}(r)=\frac{1}{N-m}\sum_{j=1,j\neq i}^{N-m+1}\Theta (r-d(X_{i}^{m},X_{j}^{m}))$$

with Θ being the Heaviside step function and *r* being the tolerance threshold. *r* is equal to 0.2 standard deviation of the EMG signals.

Then, calculate the average of $B_{i}^{m}(r)$ across all vectors $X_{i}^{m}$:

(11) $$B^{m}(r)=\frac{1}{N-m+1}\sum_{i=1}^{N-m+1}B_{i}^{m}(r)$$

Repeat the above steps to obtain the average $B_{i}^{m+1}(r)$ with the embedded dimension *m* + 1:

(12) $$B^{m+1}(r)=\frac{1}{N-m}\sum_{i=1}^{N-m}B_{i}^{m+1}(r)$$

Lastly, the sample entropy is defined as:

(13) $$SampEn(m,r,N)=-\ln\left(\frac{B^{m+1}(r)}{B^{m}(r)}\right)$$

After extracting the above features to comprise the feature vectors for individual sliding windows, the samples were divided into two categories according to the corresponding training phase. That is, the samples from the flexion phase of both experiments were considered as the painless samples, and the samples from the joint hold phase were categorized into the pain samples. Although the joint flexion phase contained samples corresponding to pain levels at 1 or 2, the samples were termed painless samples for ease of presentation.

#### 2.2.3. Estimation of maxAP via Pain State Classification 

The estimation of the maxAP was realized via the binary classification of pain states between the pain and painless samples, and two different classifiers were tested in this study, including the support vector machine and random forest model using the data from Experiment II. 

Support vector machine (SVM): In this study, the cost-sensitive support vector machine (CSVM) was selected as the classifier that can map the feature vector V to the category label y=±1, with −1 representing the painless samples and 1 representing the pain samples. Given the feature vectors $V_{i}\in R^{n}$ and category labels $y_{i}\in \{-1,1\}$, CSVM solves the following optimization problem:

(14) $$\begin{array}{ll} & \underset{\omega ,b,\xi }{\min}\;\frac{1}{2}\omega^{T}\omega +RC\sum_{\left\{i|y_{i}=1\right\}}\xi_{i}+C\sum_{\left\{j|y_{j}=-1\right\}}\xi_{j}\\ \mathrm{subject}\;\mathrm{to} & y_{i}(\omega^{T}\phi (V_{i})+b)\geq 1-\xi_{i},\;\xi_{i}\geq 0,i=1,2,\ldots ,l \end{array}$$

where ϕ(⋅) maps $V_{i}$ to a higher-dimensional space and the linear basis function was selected in this study. *C* is the trade-off between classification margin and misclassified or inseparable samples, *R* is the cost factor between false negatives and false positives, $\xi_{i}$ represents slack variables, and ω is the normal vector to the hyperplane. LIBSVM [30] was used to conduct this optimization procedure. 

**Figure 3:**
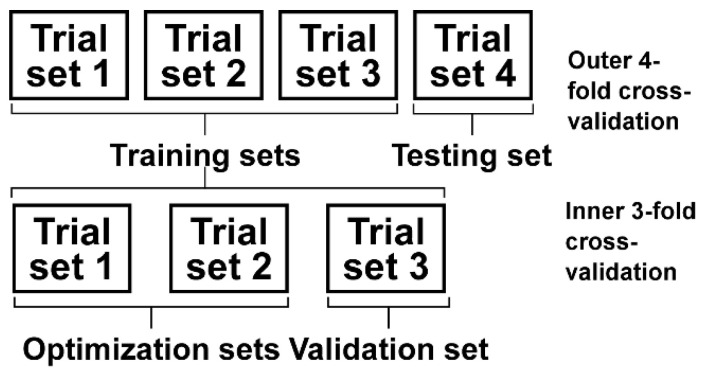
Procedures of the double cross-validation.

In Equation (14), the parameters *R* and *C* need to be determined in advance. In order to avoid an in-sample optimization problem, the double cross-validation [31,32] technique was used in this study (Figure 3). The outer cross-validation was a 4-fold cross-validation that was mainly performed to test the classification performance between the pain and painless samples. For each subject, all the trials were randomly divided into 4 sets, and each set contained two trials. One of the four sets was randomly selected as the testing set, and the other three sets were selected as the training sets. The parameters *R* and *C* were optimized using the inner 3-fold cross-validation. One of the three training sets was selected as the validation set, and the other two sets were selected as the optimization sets. The inner cross-validation was first performed with *R* and *C* varying within $\left[2^{-20},\;2^{-19},\ldots ,2^{0},\ldots ,2^{20}\right]$ independently. For each combination of parameters *R* and *C*, an evaluation criterion termed the *T* value was calculated as:

(15) $$T=0.2Acc_{v}+0.2Acc_{o}+0.3Pre_{v}+0.3Rec_{v}$$

where $Acc_{v}$ and $Acc_{o}$ were the accuracy of the validation set and optimization set, respectively; $Pre_{v}$ and $Rec_{v}$ were the precision and recall of the validation set, respectively. The accuracy, precision, recall, and F1-score were defined as:

(16) $$Acc=\frac{TP+TN}{TP+TN+FP+FN}$$

(17) $$Pre=\frac{TP}{TP+FP}$$

(18) $$Rec=\frac{TP}{TP+FN}$$

(19) $$F1=\frac{2Rec\cdot Pre}{Rec+Pre}$$

where *TP*, *TN*, *FP*, and *FN* were the number of true positives, true negatives, false positives, and false negatives, respectively. The above procedure was repeated three times until each training set had been selected as the validation set once. The *R* and *C* combination corresponding to the maximum *T* value averaged across all 3 folds was selected for the outer cross-validation. With the optimized *R* and *C* value, the CSVM model was trained using all the samples in the training sets and then tested using the testing set. All the procedures were repeated 4 times until all four sets of the outer cross-validation had been selected as the testing set once. The classification performance was evaluated using the average accuracy, precision, recall, and F1-score values across all 4 folds of the outer cross-validation.

Random forest (RF): RF is a supervised classification algorithm based on ensemble learning. It achieves high-precision classification by constructing multiple decision trees and aggregating their prediction results. With advantages in resisting overfitting, handling high-dimensional data, and parallel computing capabilities, it has been widely applied in pattern recognition tasks for EMG signals [33]. The core concept of RF involves randomly selecting subsets of the dataset to train multiple decision trees. These decision trees make predictions independently, then determine the final classification or regression output through voting or averaging. This ensemble approach reduces the risk of overfitting, as each tree is trained on different data subsets, thereby enhancing the model’s generalization capability. Some key parameters that can significantly affect the RF classification performance, including the number of decision trees *N_T_* and the minimum number of leaf nodes *N_L_*, were optimized using the training set as in the SVM method via Bayesian optimization [34]. Then, the optimized *N_T_* and *N_L_* were used to train the random forest model, which was then tested on the testing set. As in the SVM method, all steps were repeated 4 times until every set of the outer cross-validation had been selected as the testing set once. The classification performance was evaluated using the average accuracy, precision, recall, and F1-score values across all 4 folds of the outer cross-validation.

## 3. Result

### 3.1. EMG Features

**Figure 4:**
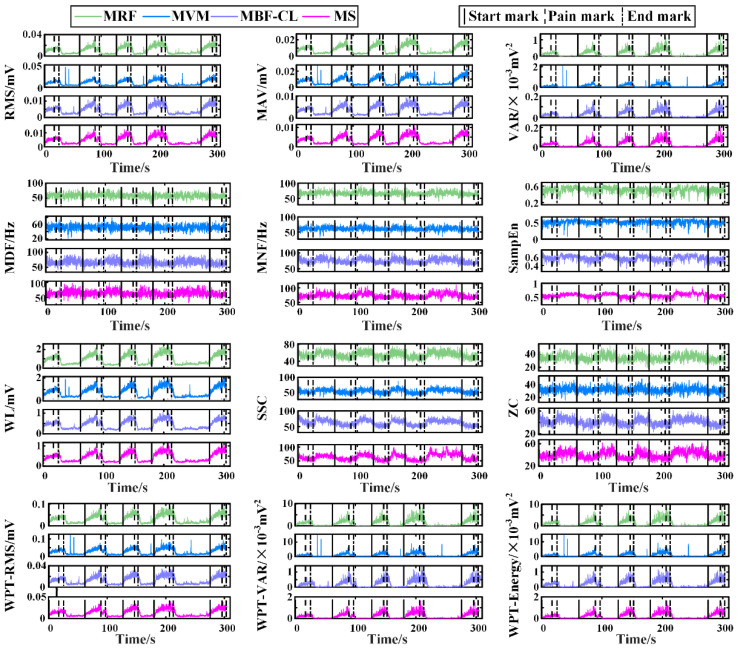
The time series of individual features from a representative subject of Experiment I.

Figure 4 illustrates the time series of individual features from a representative subject in Experiment I. For the wavelet packet features, the RMS, VAR, and energy calculated using the coefficients corresponding to the first sub-node of the third decomposition level are illustrated here. The RMS, MAV, VAR, WL, and wavelet packet features including the WPT-RMS, WPT-VAR, and WPT-Energy demonstrated a consistent trend, that is, first increasing slowly during the joint flexion phase and then plateauing during the joint hold phase, which basically indicated increased muscle activation with increased pain level. Also, it is interesting to find that not only the extensors, i.e., the MRF and MVM muscles, but also the flexors, i.e., the MBF-CL and MS muscles, showed increased EMG activity during knee flexion. However, compared with the flexors, the extensors had larger EMG amplitude and power, which indicated more activated extensors compared with the flexors, possibly leading to the final extension force to resist the flexion of knee joints. The change in the frequency domain features including the MDF, MNF, SSC, and ZC and the nonlinear feature SampEn over time was indistinct compared with the other features mentioned above. It should be noted that the feature SSC and ZC characterize the frequency domain information of the EMG activity, although they are calculated from the time–domain waveform.

**Figure 5:**
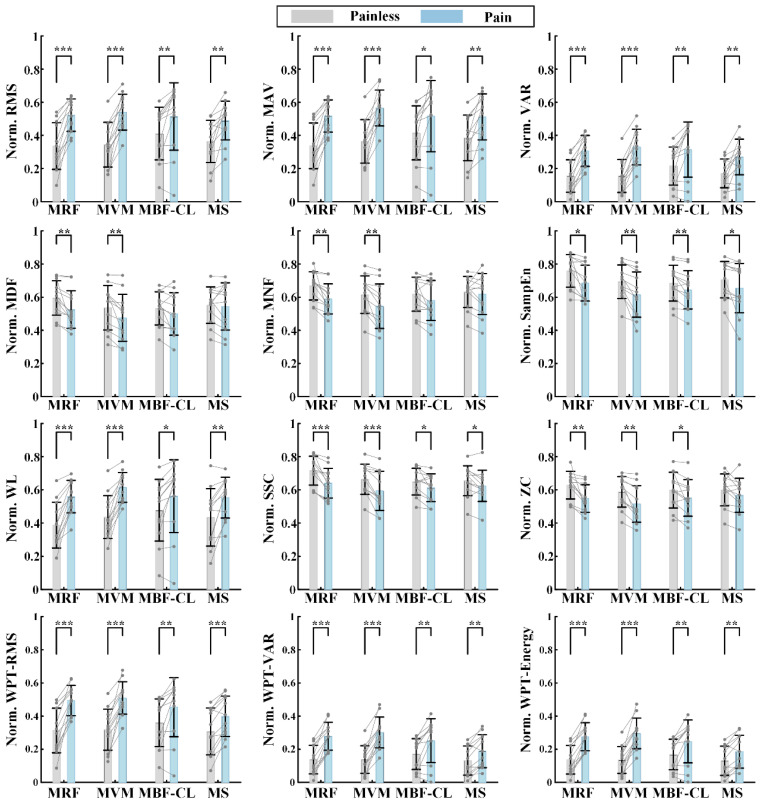
The comparison of the features between the pain and painless state for individual muscles in Experiment I. * *p* < 0.05, ** *p* < 0.01, *** *p* < 0.001.

In order to further verify whether pain state can affect EMG activity in a consistent manner across subjects, the features from Experiment I were first normalized and then averaged across the painless samples and pain samples, respectively, for individual subjects. The comparison of the features between the two states is illustrated in Figure 5 for individual muscles using the paired t-test. Overall, almost all the features showed significant differences between the two states, expect for the frequency domain features MDF and MNF of the two flexors and the ZC feature of the MS muscle. Meanwhile, the differences in the extracted features between the painless and pain state were more pronounced in the extensors compared with the flexors. Lastly, the differences in the features reflecting the amplitude information EMG signals between the two states were more pronounced compared with the features characterizing the frequency domain information. The results demonstrated that the pain level associated with the knee joint position can affect the extracted EMG features, indicating the feasibility of pain state classification for maxAP estimation. 

### 3.2. Pain State Classification for maxAP Estimation

The classification performance between painless and pain samples quantified using accuracy, precision, recall, and F1-score using the SVM and RF models is illustrated in Table 2 and Table 3, respectively, for individual subjects. The average accuracy, precision, and recall are 87.90% ± 4.55%, 80.03% ± 8.01%, and 94.39% ± 3.21%, respectively, resulting in an average F1-score of 86.38% ± 4.66% for the SVM method. In comparison, the RF method obtained a higher precision (84.49% ± 6.71%) and accuracy (89.10% ± 4.39%) while obtaining a lower recall (89.73% ± 4.38%), indicating that there were more false negatives and less false positives. A further paired t-test showed that there were significant differences in the accuracy, precision, and recall values between the SVM and RF methods (accuracy, t(6) = −2.7771, *p* = 0.0321; precision, t(6) = −4.1177, *p* = 0.0062; recall, t(6) = 3.1507, *p* = 0.0198). However, the F1-score showed no significant differences (t(6) = −1.3522, *p* = 0.2251). In order to determine whether a subset of the extracted EMG features could obtain an improved performance, a sequential forward selection of features was performed, and the resultant classification performance is illustrated in Supplementary Materials A. The results demonstrate that a subset of the EMG features would not improve the classification performance.

**Figure 6:**
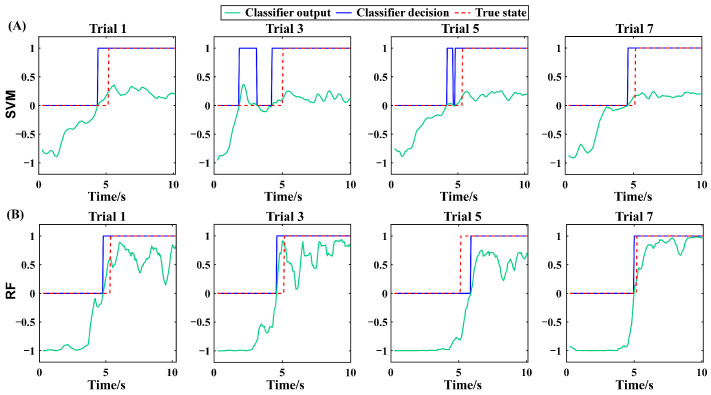
The SVM output and decision of the four odd trials from a representative subject of Experiment II (**A**). The RF output and decision of the four odd trials from a representative subject of Experiment II (**B**). The results are all from the testing set.

In Experiment II, the rehabilitation robot assisted to flex the knee joint to the maximum flexion angle position, i.e., maxAP, where the pain level just reached three. This procedure mimicked the realistic condition during rehabilitation robot-based joint mobility training. In an ideal scenario, the proposed method is expected to precisely predict the transition of the pain state. This requires the classifier to achieve a 100% classification accuracy. However, since such perfect performance is unattainable at the current stage, from a safety perspective, we prioritize the early over the delayed prediction of the pain state transition. This preference stems from the fact that premature prediction may only indicate inadequate training, whereas delayed prediction could imply that the joint mobility training has exceeded the currently safe threshold, thereby potentially leading to secondary injuries. Figure 6 illustrates the outputs of the SVM and RF methods in the four odd trials from a representative subject in Experiment II. The output was smoothed to remove the sporadic and isolated false positives and false negatives. With the red dashed curve indicating the moment of pain state transition, the SVM and RF output showed a similar pain state prediction pattern across trials. That is, the classifier’s output was negative at the beginning of a trial and slowly increased above zero, resulting in the decision of a painless and pain state, respectively. For the SVM method, there are false positives in all four trials ahead of the transition in pain state, leading to premature prediction of the pain state. For the RF method, similar to the SVM method, the pain state was also predicted prematurely in Trial 1, 3, and 7. However, the lead time of pain state prediction was smaller compared with the SVM method. In Trial 5, the pain state was predicted hysteretically due to the false negatives after the moment of pain state transition. 

**Figure 7:**
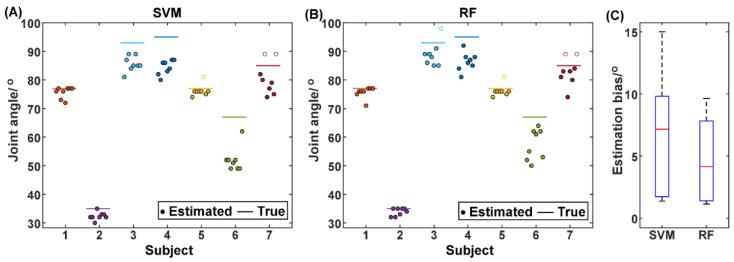
The joint angle when the pain state was first detected for individual trials of individual subjects using the SVM (**A**) and RF (**B**), respectively. The hollow circles represent the trials where the first pain detection occurs during the joint hold phase. The comparison of the estimation bias of the joint angle between the SVM and the RF methods (**C**).

As mentioned above, the proposed method is expected to predict the pain state prior to and as close as possible to the transition moment of the pain state. From the perspective of detecting maxAP, this is equivalent to the proposed method being able to underestimate the maxAP while keeping the bias as small as possible. In order to verify the performance of maxAP estimation via the pain state classification, the joint angle corresponding to the timing when the state was predicted to convert from the painless state to the pain state was extracted for the first time for each trial and is illustrated in Figure 7. For the SVM method, among the 56 trials from all subjects, only three trials from Subject 5 and 7 obtained the delayed prediction of pain state (hollow circles in Figure 7). In contrast, four trials from Subject 3, 5, and 7 obtained the hysteretic prediction of pain state. It should be noted that the distance between the hollow circles and the corresponding true maxAP lines does not represent the estimation bias of joint angles, because the rehabilitation robot would stop further flexing when the maxAP was reached. When observing the left trials that predicted the pain state prior to the transition moment of the pain state, the RF classifier had a smaller estimation bias between the estimated and actual maxAP, manifested as the solid circles having smaller distances to the true maxAP lines. The estimation bias was further averaged across trials for individual subjects and is illustrated in Figure 7C. On average, compared with the SVM method that obtained an estimation bias of 6.5° ± 5.1° across subjects, the RF method decreased the average bias to 4.5° ± 3.5°. A further paired t-test showed that the performance improvement was significant (t(6) = 2.8167, *p* = 0.0305).

## 4. Discussion

For post-fracture rehabilitation training, dynamic monitoring of the maximum angle position (maxAP) in patients’ stiff knee joints is the key to achieving rehabilitation robot-based adaptive mobility training. This approach ensures that each time of joint flexion/extension mobility training can fully stretch the adhered soft tissues while maintaining safety. In this study, a joint maxAP estimation method via pain state classification using unconscious EMG activities during flexion of stiff knee joints was proposed for potential use in post-fracture robot-based rehabilitation training. The basic assumption was that when joint flexion causes pain sensation due to joint stiffness, muscle contraction would be induced unconsciously to resist joint flexion in order to prevent further increases in pain. This hypothesis was first verified through Experiment I, in which the rehabilitation therapist assisted the patients in performing routine exercise training of stiff knee joints and the EMG signals were recorded simultaneously. The results showed that the EMG features varied with the pain level changing from 0 to 3 (Figure 4), and further statistical analysis (Figure 5) of the extracted EMG features between the painless (pain level at 0, 1 and 2) and pain (pain level at 3) states showed that the EMG activity variation induced by pain sensation was consistent across subjects for most of the commonly used EMG features, especially from the knee extensors, indicating the feasibility of classification between pain and painless states. Then, in order to further verify the pain state classification performance and the resultant maxAP detection accuracy using the EMG activities, two classifiers, i.e., SVM and RF, were tested in a pseudo-online manner on the EMG signals collected from Experiment II, in which a previously developed rehabilitation robot helped the patients to perform flexion and extension of stiff knee joints. The SVM obtained an averaged precision and recall of 80.03% ± 8.01% and 94.39% ± 3.21%, respectively, leading to an average maxAP estimation bias of 6.5° ± 5.1° across subjects, with three trials hysteretically predicting the pain state in total. In contrast, the RF method obtained an increased precision of 84.49% ± 6.71% and a decreased recall of 89.73% ± 4.38%, which helped to significantly decrease the average maxAP estimation bias to 4.5° ± 3.5°. However, the RF method also had more trials with delayed prediction of pain state. These results indicated that the unconscious EMG activity induced by pain sensation can be used to accurately classify pain states, especially during stiff knee flexion with the assistance of rehabilitation robots, thus making it possible to autonomously determine the maximum joint angle position according to the pain level for robot-based rehabilitation training, potentially facilitating the application of the robotic technique in rehabilitation after bone fracture.

In this study, a variety of EMG features were extracted for pain state classification. Although the statistical analysis showed that most features had significant differences between the two states, the features reflecting the EMG amplitude or energy were more distinct compared with the features reflecting the oscillation frequency or the complexity of EMG signals (Figure 5 and Figure 6). The increased EMG amplitude with pain indicated a higher muscle activation level and was consistent with the previous studies that utilized the EMG amplitude information to explore the pain-induced EMG activity [25,26,35,36]. The MDF, MNF, SSC, and ZC features diminished, indicating a slower oscillation of EMG waveforms under the pain state compared with the painless state. Recently, a study investigated the influence of acute pain on motor unit discharge properties, and the results showed that the discharge rate of quadriceps motor units was lower during the pain condition than the control condition [24], possibly explaining why the oscillation of EMG waveforms became slower under the pain state in this study. 

Furthermore, the study [24] showed that not only the pain in the antagonist biceps femoris but also the pain in the distant muscles that are unable to modify the load of the contracting quadriceps can modify the discharge properties of the quadriceps, indicating that the pain can affect the muscle activity in multiple ways except for the biological mechanisms of harm avoidance. This possibly explains why the muscle contraction strengthened in the MBF-CL and MS muscle when the flexion-induced pain occurred in the knee joint. For example, the tension of subjects can possibly induce the involuntary contraction of all muscles including the flexors of knee joint. It should be noted that despite the flexors also being activated by the pain sensation induced by knee flexion, the EMG activation level was much smaller compared with the extensors, indicating the ultimate consequence of resisting further knee flexion. In our preconceived situation, there would be a sudden muscle contraction inducing rapidly increasing EMG activity immediately after patients experience an intolerable pain level. However, our results showed that the EMG amplitude increased slowly with the flexion of knee joints. The main reason is that in both Experiment I and Experiment II, the knee joint was flexed slowly with the assistance of either the therapist or the robot. The patients therefore can clearly feel the pain level slowly increasing from 0 to 3, resulting in the slowly enhanced muscle contraction. Although, the EMG activity could possibly result from the stretch reflex activity, i.e., the activation of antagonist muscles. However, the stretch-induced EMG activity can be neglected when the joint rotational velocity is small for individuals without neurological diseases [37]. In addition to the stretch reflex, some other factors might also introduce interfering muscle activations, which could disturb the classification of pain levels, such as anxiety and the activation of synergistic muscles. In order to rule out these potential confounding factors, we recruited four healthy subjects to perform Experiment II (Supplementary Materials B). The results showed that the EMG amplitude change was much smaller compared with that of the patients, and no consistent variation in EMG activity between the flexion phase (equivalent to the non-pain state in patient experiments) and holding phases (equivalent to the pain state in patient experiments) was observed. These results indicated that compared with these physiological factors, the EMG activity induced by pain in patients dominates during the flexion of the stiff knee joint, which ensures the accuracy of using EMG signals for pain level classification.

In this study, the conventional time/frequency domain features and nonlinear features were extracted to distinguish between the two pain states. Among these features, some are relatively similar and reflect the same aspect of the EMG activity. For instance, RMS and MAV both capture the amplitude information of EMG signals. However, due to their different assumptions on the underlying distribution of EMG data [38], they may be applicable to different individuals. In our preliminary test, the sequential forward selection of features was performed in order to reduce possible information redundancy and enhance classification performance. However, the results (Supplementary Materials A) indicated that the feature selection did not improve the classification performance. One possible explanation is that, despite the similarity between some features, they actually reflect distinct details of the EMG activity, each contributing to pain state classification. On the other hand, most features exhibit clear monotonic increases or decreases with the pain level changing from 0 to 3. The binary pain state recognition is fundamentally a threshold selection issue, and its performance relies on the stability of the relationship between the feature magnitudes and the pain level. For example, a feature that would always reach a specific fixed magnitude when the pain level repeatedly reaches three can result in a high classification performance. Features that appear redundant may mitigate the susceptibility of single features to interference from noise or other factors, thereby ensuring—on average—the stability of the relationship between feature magnitudes and pain intensity. This could be another reason why reducing feature dimensionality did not alter the classification performance.

When the proposed pain state classification method is used in a fracture rehabilitation robotic system, it is expected to be able to predict the pain state beforehand while being as close as possible to the timing of the joint reaching its maximum angle position, corresponding to as specific a pain level as possible. The former guarantees that the robot would not bring the secondary damage to the joint or too much discomfort to patients, and the latter aims at obtaining adequate training for each trial. In Experiment II with the rehabilitation robot assisting knee joint flexion, it is encouraging to find that the pain state was predicted before the joint angle reached its maxAP for 94.64% and 92.86% of trials using the SVM and RF method, respectively. This may result from the fact that the mean recall was higher than the precision, indicating that there were more false positives (painless sample misclassified into pain samples) than false negatives (pain samples misclassified into painless samples). Meanwhile, with the sporadic and isolated false positives and false negatives removed via smoothing, more false positives that can lead to the final decision of classifiers were retained near the pain state transition moment. These together lead to the early prediction of pain state for both SVM and RF classifiers. In addition, compared with the SVM method, the RF method obtained an increased precision and a decreased recall and therefore made the prediction timing of pain state closer to the pain state transition moment. This consequently led to a small estimation bias of the maxAP and possibly more trials with hysteretic pain state prediction. As for why there were typically false positives before reaching the maximum joint angle, the major reason was that the muscle contraction strength manifested because, for example, the EMG amplitude generally plateaued before reaching the target joint angle. It should be noted that the maximum joint angle was determined by patients’ self-reporting on pain levels, and unconscious muscle contraction might precede patients’ reporting due to the reaction delay.

The role of pain level monitoring is manifested in two ways. Firstly, the pain level needs to be monitored to reach some specific level in order to stretch the intra-articular adhesive tissues adequately. Secondly, the pain level that can potentially lead to further damage should be avoided. From the perspective of safety, the latter is more important than the former. As shown in Figure 7, the proposed pain classification method predicted the pain state before reaching the maxAP in most cases, which can guarantee the safety of patients’ pathological knee joints. However, we have to admit that there were still trials in which the pain state was not predicted before reaching the maxAP, which could potentially bring damage to patients’ knee joints. Therefore, in our exoskeleton knee robotic system, the pain classification framework needs to be combined with some adaptive interactive control strategy in order to avoid the possible damage to patients. In fact, our preliminary test had shown that the motor load force can reflect pain levels. This occurs because when the stiff knee joint moves to its maximum ROM, adhesive tissues restrict further movement, simultaneously increasing pain intensity. In our future study, the human–robot interaction forces will be combined with EMG signals in the actual control of rehabilitation robots via, for example, the fuzzy logic control, allowing for more accurate pain state detection and safe adaptive rehabilitation training. 

The pain level at three was developed based on clinicians’ experience to ensure full release of intra-articular adhesions without causing secondary injury to the stiff joint. It might be concerning that at low pain levels, patients may struggle to differentiate between adjacent levels due to subtle differences. However, it should be noted that since pain is a complex subjective psycho-physiological experience lacking objective measurement methods and pain tolerance varies significantly among individuals, our study does not necessitate reaching precisely level three. Rather, we aim for patients to consistently achieve a personally tolerable pain level that remains relatively constant across experimental trials.

As mentioned above, one limitation of this study is that the proposed maxAP detection method was not tested in the closed-loop control of the fracture rehabilitation robotic system for adaptive joint mobility training. In our further study, the pain classification-based maxAP estimation method will be embedded into the previously developed knee fracture rehabilitation robots [23] to verify its safety and effectiveness in improving the ROM of stiff knee joints. The other limitation was that the extracted EMG features were all conventional global time/frequency domain features of EMG signals, and a further study will explore other features that can possibly further improve the pain state classification performance. For example, the previous study [24] has demonstrated that the motor unit discharge rate is influenced by the pain sensation, and the online EMG decomposition technique [39,40] could provide an effective tool to monitor the motor unit discharge rate and then the pain state. 

## 5. Conclusions

In this study, a pain state classification method was proposed to estimate the maximum joint angle position for the adaptive mobility training of fracture-induced stiff joints in rehabilitation robotic systems. The feasibility of using unconscious EMG activities to classify the pain state and then estimate the maximum angle position during flexion of stiff knee joints was investigated. The results showed that the pain state can significantly affect the extracted time/frequency domain features and nonlinear features, and the binary classification performance between the pain and painless states obtained an accuracy of 89.10% ± 4.39%, resulting in an average estimation bias of 4.5° ± 3.5° of the maximum angle position using the random forest classifier. These preliminary results showed that the pain state can be recognized accurately by solving the binary classification problem, which can be further used to autonomously determine the optimal angle position for joint mobility training. The proposed method can potentially facilitate the application of the robotic technique in rehabilitation after bone fracture. 

## Figures and Tables

**Table 1:** Demographic information of subjects.

Patient	Age	Gender	Weight (Kg)/Height (cm)	Fracture Site	Post-Operation (Week)
**Experiment I**					
1	50	M	64/173	Meniscus	11
2	61	F	62/170	patella	35
3	30	M	66/175	patella	6
4	24	F	61/168	tibia	12
5	17	F	62/170	patella	8
6	41	M	67/175	medial femoral condyle	8
7	62	F	62/160	patella	7
8	16	F	54/160	patella	8
9	40	F	59/160	patella	13
10	35	M	85/180	tibia	1
11	52	F	60/160	patella	12
12	32	F	55/165	tibia	12
**Experiment II**					
1	32	F	110/160	meniscus	1
2	47	F	130/170	patella	9
3	35	M	65/175	tibia	7
4	61	F	58/163	patella	12
5	51	M	75/175	tibia	3
6	51	F	60/165	tibia	8
7	50	F	55/170	patella	12

**Table 2:** SVM classification performance.

Subject	Accuracy	Precision	Recall	F1-Score
1	92.18%	89.54%	95.57%	92.45%
2	86.59%	84.61%	93.09%	88.64%
3	86.09%	78.76%	95.33%	86.25%
4	80.32%	70.15%	99.15%	82.16%
5	93.77%	87.66%	94.95%	91.15%
6	86.07%	69.25%	94.24%	79.83%
7	90.28%	80.26%	88.46%	84.16%
Mean ± std	87.90% ± 4.55%	80.03% ± 8.01%	94.39% ± 3.21%	86.38% ± 4.66%

**Table 3:** RF classification performance.

Subject	Accuracy	Precision	Recall	F1-Score
1	91.43%	88.36%	95.47%	91.78%
2	89.58%	90.53%	90.98%	90.75%
3	87.40%	84.80%	88.30%	86.51%
4	80.96%	73.81%	90.46%	81.29%
5	94.97%	91.21%	94.20%	92.68%
6	87.99%	76.88%	84.34%	80.44%
7	91.38%	85.84%	84.36%	85.09%
Mean ± std	89.10% ± 4.39%	84.49% ± 6.71%	89.73% ± 4.38%	86.94% ± 4.98%

## Data Availability

The data that support the findings of this study are available upon reasonable request from the authors.

## Supplementary Materials

The following supporting information can be downloaded at: https://www.mdpi.com/article/10.3390/s25165142/s1, Figure S1: The original EMG signals of the MRF, MVM, MBF-CL and MS muscles from a representative healthy subject participating in Experiment II (A) and the comparison of the normalized EMG amplitude between the flexion and holding phase for individual muscles (B); Table S1: SVM classification performance after feature sequential forward selection; Table S2: RF classification performance after feature sequential forward selection.
