# Supplementary material for: Pain State Classification of Stiff Knee Joint Using Electromyogram for Robot-Based Post-Fracture Rehabilitation Training
Source: Sensors (Basel). 2025 Aug 19;25(16):5142. doi: 10.3390/s25165142 (PMC12389886; doi:10.3390/s25165142)
Supplement: Supplementary file 1 [file sensors-25-05142-s001.zip › sensors-3740097-supplementary.pdf]

Supplementary Materials A

Table S1. SVM classification performance after feature sequential forward selection.

Subject	Accuracy	Precision	Recall	F1-score
1	92.79%	89.36%	97.18%	93.10%
2	88.23%	86.49%	93.69%	89.94%
3	86.66%	79.91%	94.68%	86.67%
4	80.59%	70.82%	97.89%	82.18%
5	94.05%	89.20%	93.74%	91.41%
6	85.89%	69.87%	91.06%	79.06%
7	90.14%	79.04%	90.08%	84.19%
Mean±std	88.33%±4.56%	80.67%±8.15%	94.04%±2.88%	86.65%±5.14%

Table S2. RF classification performance after feature sequential forward selection.

Subject	Accuracy	Precision	Recall	F1-score
1	90.72%	87.64%	94.87%	91.11%
2	88.96%	89.94%	90.48%	90.21%
3	87.72%	85.01%	88.85%	86.89%
4	80.11%	72.95%	89.80%	80.50%
5	95.16%	91.78%	94.10%	92.93%
6	87.93%	76.81%	84.18%	80.33%
7	90.99%	85.06%	83.86%	84.46%
Mean±std	88.80%±4.59%	84.17%±6.89%	89.45%±4.31%	86.63%±5.08%

Supplementary Materials B

In order to rule out the influence of some other potential factors that can lead to the activation of muscles, such as anxiety and activation of antagonist or synergistic muscles, four healthy subjects were recruited to perform Experiment II. Since they had no pain issues at the knee joints, the target angle was uniformly set at 80°. The EMG signals recorded from one subject are shown in Figure S1(A). It can be observed that the EMG signals from the extensors did not exhibit significant changes with joint movement, while the EMG signals from the flexors, though displaying some regularity, had low amplitudes. Moreover, the patterns and magnitudes of EMG amplitude changes differed markedly from those caused by pain reported by patients. Furthermore, we calculated the EMG signal amplitudes (root mean square) during the knee flexion phase (equivalent to the non-pain state in patient experiments) and the holding phase (equivalent to the pain state in patient experiments). The average results for the four subjects are shown in Figure S1(B). The findings indicate no consistent variation in EMG amplitude between the flexion and holding phases across subjects.

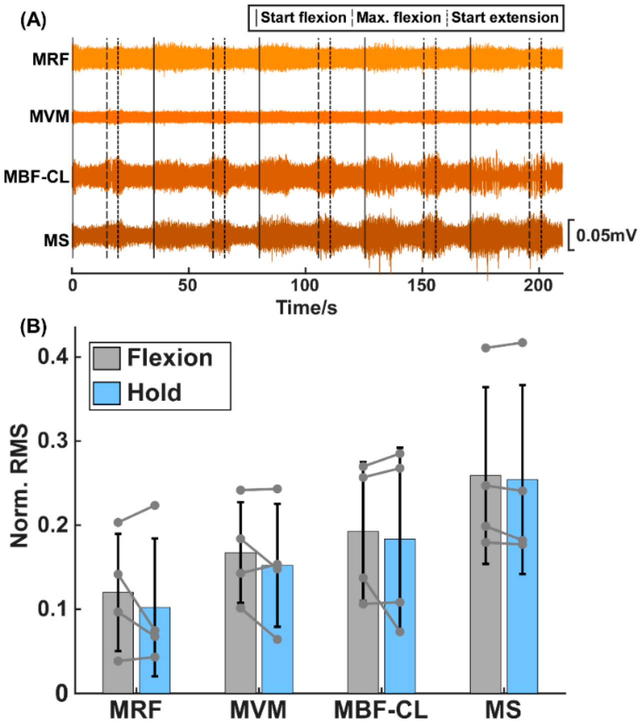

Figure S1. The original EMG signals of the MRF, MVM, MBF-CL, and MS muscles from a representative healthy subject participating in Experiment II (A) and the comparison of the normalized EMG amplitude between the flexion and holding phase for individual muscles (B).
